# Characterization of Ferredoxin-Dependent Biliverdin Reductase PCYA1 Reveals the Dual Function in Retrograde Bilin Biosynthesis and Interaction With Light-Dependent Protochlorophyllide Oxidoreductase LPOR in *Chlamydomonas reinhardtii*

**DOI:** 10.3389/fpls.2018.00676

**Published:** 2018-05-23

**Authors:** Weiqing Zhang, Huan Zhong, Hui Lu, Yuxiang Zhang, Xuan Deng, Kaiyao Huang, Deqiang Duanmu

**Affiliations:** ^1^State Key Laboratory of Agricultural Microbiology, College of Life Science and Technology, Huazhong Agricultural University, Wuhan, China; ^2^Key Laboratory of Algal Biology, Institute of Hydrobiology, Chinese Academy of Sciences, Wuhan, China

**Keywords:** PCYA1, bilin, POR, chlorophyll biosynthesis, algae, photoacclimation

## Abstract

Bilins are linear tetrapyrroles commonly used as chromophores of phycobiliproteins and phytochromes for light-harvesting or light-sensing in photosynthetic organisms. Many eukaryotic algae lack both phycobiliproteins and phytochromes, but retain the bilin biosynthetic enzymes including heme oxygenase (HO/HMOX) and ferredoxin-dependent biliverdin reductase (FDBR). Previous studies on *Chlamydomonas reinhardtii* heme oxygenase mutant (*hmox1*) have shown that bilins are not only essential retrograde signals to mitigate oxidative stress during diurnal dark-to-light transitions, they are also required for chlorophyll accumulation and maintenance of a functional photosynthetic apparatus in the light. However, the underlying mechanism of bilin-mediated regulation of chlorophyll biosynthesis is unclear. In this study, Chlamydomonas phycocyanobilin:ferredoxin oxidoreductase PCYA1 FDBR domain was found to specifically interact with the rate-limiting chlorophyll biosynthetic enzyme LPOR (light-dependent protochlorophyllide oxidoreductase). PCYA1 is partially associated with chloroplast envelope membrane, consistent with the observed export of bilin from chloroplast to cytosol by cytosolic expression of a bilin-binding reporter protein in Chlamydomonas. Both the *pcya1-1* mutant with the carboxyl-terminal extension of PCYA1 eliminated and efficient knockdown of *PCYA1* expression by artificial microRNA exhibited no significant impact on algal phototrophic growth and photosynthetic proteins accumulation, indicating that the conserved FDBR domain is sufficient and minimally required for bilin biosynthesis and functioning. Taken together, these studies provide novel insights into the regulatory role of PCYA1 in chlorophyll biosynthesis via interaction with key Chl biosynthetic enzyme.

## Introduction

Evolutionary origin of higher plants and eukaryotic algal chloroplast can be traced back to approximately 1.5 billion years ago during the primary endosymbiotic event, namely a eukaryotic cell engulfing a free-living cyanobacterium and the endosymbiont gradually evolving into modern-day photosynthetic organelle (Keeling, 2010; de Vries and Archibald, 2017). As a semi-autonomous essential organelle present in all extant eukaryotic oxygenic photosynthetic organisms, chloroplast contains the photosynthetic machinery and also serves as the factory of numerous biosynthetic pathways to provide intermediate biomolecules or end products essential for plant development and physiological functions (Joyard et al., 2009). Additionally, to sense extracellular environmental cues and integrate intracellular signals, a functional chloroplast is critical for biogenic and stress responses, which requires the coordinated expression between chloroplast and nucleus genomes (Fernández and Strand, 2008; Xiao et al., 2013). The bilateral communication is largely facilitated by anterograde and retrograde signaling pathways (Woodson and Chory, 2008; Bobik and Burch-Smith, 2015).

In the past three decades, many different retrograde signaling pathways in photosynthetic species are discovered and well characterized by using model organisms such as *Arabidopsis thaliana* and *Chlamydomonas reinhardtii* (Arabidopsis and Chlamydomonas thereafter) (Chi et al., 2013, 2015; Börner, 2017). Briefly, these retrograde signals can be cataloged into at least five distinct groups based on the sources of origin: tetrapyrrole intermediates (Terry and Smith, 2013), reactive oxygen species (ROS) (Wakao et al., 2014), plastid redox status (Bräutigam et al., 2009), plastid gene expression (Leister et al., 2017), and other chloroplast-derived metabolites such as 3′-phosphoadenosine 5′-phosphate (PAP), methylerythritol cyclodiphosphate (MEcPP) (Xiao et al., 2012; Chan et al., 2016).

Tetrapyrroles, including chlorophyll, siroheme and heme, are mainly produced in chloroplasts and share a common biosynthetic pathway starting from the precursor 5-aminolevulinic acid (ALA) (Tanaka and Tanaka, 2007; Tanaka et al., 2011). Tetrapyrroles play significant roles in many physiological processes such as photosynthesis and drought acclimation (Nagahatenna et al., 2015). Over-accumulated tetrapyrrole intermediates are highly phototoxic and can induce oxidative stress upon light illumination. Therefore, a precise regulation of tetrapyrrole biosynthesis is vital to avoid oxidative stress caused by mis-accumulation of phototoxic tetrapyrrole intermediates (Mochizuki et al., 2010; Busch and Montgomery, 2015). Tetrapyrrole intermediates are also reported to control the nuclear gene expression both positively and negatively as retrograde signals (Larkin, 2014; Brzezowski et al., 2015). Previous studies have shown that the accumulated Mg-Protoporphyrin IX (MgPPIX) acts as a retrograde signal emitting from plastid to negatively regulate photosynthetic gene expression (Strand et al., 2003; Ankele et al., 2007; Zhang et al., 2011). In contrast, a ferrochelatase 1 (FC1)-overexpression mutant in Arabidopsis (*gun 6-1D*) implied that the specific heme pool produced by FC1 was accountable for upregulation of several photosynthesis associated nuclear genes (PhANGs) expression (Woodson et al., 2011).

Heme oxygenase (HO) and ferredoxin-dependent biliverdin reductase (FDBR) could further convert heme to open-chain tetrapyrroles, i.e., biliverdin IXα (BV IXα) and bilins, respectively (Dammeyer and Frankenberg-Dinkel, 2008). Distinct FDBRs with different regiospecificities are found in photosynthetic organisms from cyanobacteria, eukaryotic algae to land plants to yield various types of bilins, such as phycocyanobilin (PCB), phytochromobilin (PΦB), phycoerythrobilin (PEB) and phycourobilin (PUB) (Rockwell et al., 2014b; Rockwell and Lagarias, 2017). These bilins usually act as cofactors of phycobiliproteins for light-harvesting in cyanobacteria and some eukaryotic algae (Singh et al., 2015), or as the chromophores of phytochromes for light sensing in many photosynthetic organisms (Falklöf and Durbeej, 2016). It is thus surprising that although some green algae lack phycobiliproteins and phytochromes, all of them contain the bilin biosynthetic enzymes including HO and certain form of FDBR (Duanmu et al., 2014). Based on characterization of the Chlamydomonas *hmox1* mutant, two recent reports proposed a more ancient and possibly widely conserved function of bilins as biogenic retrograde signals essential for photoacclimation and functional chloroplast maintenance during diurnal transition from dark to light (Duanmu et al., 2013, 2017). It was also hypothesized that chlorochrome, a putative bilin-dependent blue-light photoreceptor residing in the chloroplast, is involved in regulation of the chlorophyll (Chl) biosynthesis and photosystem I (PSI) and light-harvesting complex I (LHCI) protein accumulation (Wittkopp et al., 2017).

However, the biochemical evidence of bilin transportation and the underlying mechanism of bilin-mediated regulation of Chl biosynthesis is still unclear. In this study, we provided evidences that Chlamydomonas PCYA1 protein directly interacts with the key Chl biosynthetic enzyme LPOR (light-dependent protochlorophyllide oxidoreductase), and this interaction is specific to Chlamydomonas since the Arabidopsis homologous proteins do not interact with each other. PCYA1 is also partially associated with the chloroplast envelope and absent from thylakoid membrane. Heterologous expression of a bilin-binding reporter protein in the cytosol of Chlamydomonas confirmed that the bilin molecule could be exported from chloroplast to cytosol, an essential character of chloroplast retrograde signals. Furthermore, analysis of an insertional mutant *pcya1-1* and knockdown mutants of *PCYA1* by artificial microRNA demonstrated that loss of carboxyl-terminal extension (CTE) and reduced accumulation of PCYA1 have no significant impact on phototrophic growth and PSI related proteins accumulation in Chlamydomonas. These results provide further insights into direct regulation of Chl biosynthesis by bilin biosynthetic enzyme and putative bilin transport in photosynthetic eukaryotes.

## Materials and Methods

### Chlamydomonas Strains and Growth Conditions

*Chlamydomonas reinhardtii* wild-type strain 4A+ and *hmox1* mutant were described previously (Duanmu et al., 2013). CC400 was obtained from the Chlamydomonas Stock Center, University of Minnesota, St. Paul, United States. The *pcya1-1* mutant and its parental strain HS211 were obtained from Institute of Hydrobiology, Chinese Academy of Sciences, Wuhan, China (Cheng et al., 2017). All strains were maintained on TAP (Tris-acetate-phosphate) agar plates with a revised mineral element recipe (Kropat et al., 2011), at 22∼24°C and under cool-white fluorescent light (10∼20 μmol photons m^-2^s^-1^). For phototrophic phenotype comparison, cells were resuspended in TP (Tris-phosphate without acetate) medium, spotted on TAP or TP agar plates and maintained under dark, low light (∼60 μmol photons m^-2^s^-1^), elevated light (∼700 μmol photons m^-2^s^-1^) or dark/light (12 h dark/12 h light) diurnal conditions. For photosystem I (PSI) related protein accumulation analyses, cells were grown under similar light conditions as described previously (Wittkopp et al., 2017). Briefly, all strains were grown in TAP medium under ∼30 μmol photons m^-2^s^-1^ until reaching the mid logarithmic phase, then cells were diluted in TAP medium to a density around ∼1 × 10^6^ cells/mL. Half of the cell cultures were grown in the dark for 24 h, while the other half were grown in the dark for 12 h and then exposed to light (∼160 μmol photons m^-2^s^-1^) for 12 h. Cells were harvested and used for protein extraction.

### FDBRs Sequence Alignments

FDBRs protein sequences of *C. reinhardtii* (Cre13.g587100), *Volvox carteri* (Vocar.0001s0011), *A. thaliana* (AT3G09150), *Physcomitrella patens* (Pp3c13_10580V3), *Micromonas pusilla* CCMP1545 (58884) were obtained from Phytozome 12^1^. FDBRs sequences from *Synechococcus* sp. PCC 7002 (GI: 169886494), *Synechocystis* sp. PCC 6803 (GI: 499176294), *Paulinella micropora* (APP88044.1), *Cyanidioschyzon merolae* strain 10D (GI: 544211022), *Gloeochaete wittrockiana* SAG46.84 were based on previous paper (Rockwell et al., 2017). CLUSTAL X^2^ and DNAMAN^3^ were used for multiple sequence alignments.

### Yeast Two-Hybrid Analyses of Protein–Protein Interaction

For autoactivation activity test and cDNA library screening, we followed the instruction of “HybriZAP-2.1 Two-Hybrid Libraries” system (Agilent). Coding regions of CrPCYA1ΔTP (amino acids 56–556), CrPCYA1-NTE (amino acids 56–174), CrPCYA1-FDBR (amino acids 175–451), CrPCYA1-CTE (amino acids 449–556) were amplified by PCR (see **Supplementary Table S1** for primers details) from a Chlamydomonas cDNA library described previously (Wang and Spalding, 2006) and cloned into pBD-GAL4 vector. Transformed YRG2 yeast cells containing respective recombinant constructs were maintained on synthetic dropout plates lacking tryptophan (SD-Trp) or lacking both tryptophan and histidine (SD-Trp-His) for autoactivation test. For cDNA library screening, the bait construct (pBD-CrPCYA1-FDBR) and cDNA plasmids library were sequentially transformed into YRG2 cells. The transformed yeast cells were spreaded on selective SD plates without tryptophan, leucine and histidine (SD-Trp-Leu-His) and incubated at 28°C for 2–4 days until colonies appeared, which were selected as potential interacting candidates.

To confirm the interaction between the bait and putative prey proteins, we used the Matchmaker Gold Yeast Two-Hybrid System (Clontech) following the recommended instruction manual. The coding regions of FDBR (amino acids 175–451) and LPOR (amino acids 35-397) were amplified from pBD-CrPCYA1-FDBR vector and a Chlamydomonas cDNA library, respectively. Coding regions of CHLB/CHLL/CHLN (full length of each gene) were amplified from wild-type 4A+ genomic DNA. An Arabidopsis cDNA library was used to amplify AtHY2ΔTP (amino acids 46–329), AtPORAΔTP (amino acids 54–405), AtPORBΔTP (amino acids 44–401) and AtPORCΔTP (amino acids 67–401). The bait sequences, FDBR and AtHY2ΔTP, were cloned into pGBKT7 vector and introduced into Y2H Gold yeast strain. The prey sequences were cloned into pGADT7 vector and introduced into Y187 yeast cells. To verify the bait-prey interaction, equal amount of Y2H Gold and Y187 cells were mated before spotting on SD/-Trp-Leu, SD/-Trp-Leu-Ade-His and SD/-Trp-Leu plates supplemented with 80 μg/ml β-X-gal (BIOSHARP) and incubated at 28°C for 2–3 days. Interactions of P53 with SV40, and Lam with SV40, were respectively used as positive and negative controls.

### Pull-Down Assay

Ferredoxin-dependent biliverdin reductase coding region was PCR-amplified from pGBKT7-FDBR and cloned into pGEX-6P-1 (GE Healthcare Life Sciences) to generate pGEX-6P-FDBR for GST-FDBR fusion protein expression. Similarly, coding region of LPOR was obtained from pGADT7-LPOR by PCR and cloned into vector pMAL-C2X (New England Biolabs) to produce pMAL-C2X-LPOR for expressing MBP-LPOR recombinant protein. These constructs were introduced into *Escherichia coli* BL21 for protein induction with 0.3 mM IPTG (isopropyl β-D-1-thiogalactopyranoside) at 28°C for 4 h. Cells were harvested, rinsed with 1 × PBS (135 mM NaCl, 2.7 mM KCl, 2 mM NaH_2_PO_4_, and 10 mM Na_2_HPO_4_, pH 7.4) buffer and disrupted by high pressure homogenization (D-3L; PhD Technology International, MN, United States). Cells were clarified by centrifuging at 10,000 × *g*, 10 min at 4°C and supernatants containing fusion proteins were collected. Equal volume of supernatant containing GST-FDBR or MBP-LPOR were mixed and incubated with MBP beads (New England Biolabs) at 4°C for 1–2 h. After incubation, MBP beads were collected and washed ten times with 1 × PBS buffer to remove unbound proteins, and then boiled with 1 × SDS loading buffer [50 mM Tris–HCl, pH 6.8, 2% (w/v) SDS, 0.1% (w/v) bromophenol blue, 10% (v/v) Glycerol, 1% (v/v) β-mercaptoethanol] for 10 min. The supernatant was then subjected to immunoblot analysis using GST and MBP antibodies.

### Split-Luciferase Complementation

The split-luciferase complementation assay was performed based on the previous paper (Chen et al., 2008). Briefly, coding regions of PCYA1 (full-length), FDBR and AtHY2ΔTP were cloned into JW772-cLUC vector, whereas the coding regions of LPOR, CHLB, CHLL, CHLN, AtPORAΔTP, AtPORBΔTP, and AtPORCΔTP were ligated into JW771-nLUC vector. These constructs were introduced into Agrobacterium strain GV3101 by electroporation. Logarithmic phase cells of GV3101 containing respective plasmids were centrifuged at 10,000 × *g* for 5 min, washed twice with ddH_2_O, and then resuspended in infiltration buffer (10 mM MES, pH 5.8; 10 mM MgCl_2_, 150 μM acetosyringone). After 2 h incubation at room temperature, equal amounts of Agrobacteria cells containing JW771- or JW772- vectors were mixed and co-infiltrated into *Nicotiana benthamiana* leaves. After 48 h, tobacco leaves infiltrated with bacteria were sprayed with 1 mM luciferin substrate (Gold Bio) and luminescence signals were acquired by a CCD imaging apparatus (Lumazone Pylon2048B).

### Subcellular Fractionation

The cell wall-deficient Chlamydomonas strain CC400 was used to isolate chloroplast soluble fraction, chloroplast envelope and thylakoid membrane. Cells were grown under synchronous condition (12 h light: 12 h dark) in minimal medium until reaching cell density around ∼5 × 10^6^ cells/ml. At 4th hour in the light phase, cells were harvested and centrifuged at 4,000 × *g*, 10 min and intact chloroplasts were isolated following the protocol described previously (Mason et al., 2006). Sucrose buffer A (50 mM HEPES-KOH, pH 7.5; 2 mM MgCl_2_) and sucrose buffer B (50 mM HEPES-KOH, pH 7.5; 10 mM EDTA) were prepared before the following fractionation procedures. The intact chloroplast was resuspended in sucrose buffer A at chlorophyll concentration around 500 mM. The complete lysis was monitored under microscope. Chloroplast suspension was applied on top of a gradient of sucrose buffer A (10 ml of 0.9 M sucrose and 5 ml of 0.6 M sucrose) and centrifuged at 100, 000 × *g* (SW41 Ti rotor) for 1 h. Soluble chloroplast proteins are in the sample zone (top layer, no sucrose). Chloroplast envelope membranes could be recovered as a yellow band at the interface between two sucrose layers, whereas thylakoid membranes became a pellet. Envelope membranes were collected with a Pasteur pipette, diluted with sucrose buffer A and centrifuged at 100,000 × *g* (Ti60 rotor) for 1 h. The chloroplast soluble fraction was further centrifuged at 100,000 × *g* (Ti60 rotor) for 1 h to get rid of any pellet contamination and only kept the supernatant. Thylakoid membranes were resuspended in 1.8 M sucrose buffer B. Layered on top are 1.3, 0.9, and 0.6 M of sucrose buffer B. After centrifuge at 100,000 × *g* (SW41 Ti rotor) for 1 h, purified thylakoid membranes are collected from the interface of 1.8 and 1.3 M sucrose layers. Equal volume of 0 M sucrose buffer B is added to the collected thylakoid membranes and centrifuge at 100,000 × *g* (Ti60 rotor) for 1 h to yield pellet of purified thylakoid membranes. Pelleted envelope membrane and thylakoid membrane were dissolved in SDS sample buffer [50 mM Tris–HCl, pH 6.8; 2% (w/v) SDS]. Chloroplast soluble fraction were precipitated using SDS-methanol-chloroform method as described previously (Duanmu et al., 2013).

### DtenPHY1 Expression and Protein Purification

The photosensory core module (PCM) region of *Dolichomastix tenuilepis* phytochrome DtenPHY1 was amplified from pBAD-DtPHYΔL (Duanmu et al., 2014) and ligated into an engineered vector containing the PSAD promoter and a Twin-Strep-tag. The PSAD-DtenPHY1 construct was linearized by KpnI/BamHI double digestion before glass bead transformation into Chlamydomonas wild-type strain CC400. Transgenic cells with highest DtenPHY1 protein expression were cultured in 2 L TAP medium until reaching a density of ∼5 × 10^6^ cells/mL under 60 μmol photons m^-2^s^-1^ fluorescent white light. Cells were centrifuged at 4,000 × *g* for 10 min at 4°C and resuspended in 20 mL lysis buffer (100 mM Tris–HCl, pH 8.0, 150 mM NaCl, 1 mM PMSF, 10 mM β-mercaptoethanol, 1% (v/v) Triton X-100 and 1 mM EDTA). Cells were disrupted by high pressure homogenization (D-3L; Ph.D. Technology International, MN, United States) and then clarified by centrifuge at 4,000 × *g* for 10 min at 4°C. The supernatant was collected and incubated with strep-tag resin (IBA Lifesciences) at 4°C for 2 h. Subsequently, the resin with bound proteins was washed five times with washing buffer (100 mM Tris–HCl, pH 8.0; 150 mM NaCl, 1 mM EDTA). The target protein was eluted from the resin by elution buffer (2.5 mM desthiobiotin in washing buffer) and dialyzed overnight [10% (v/v) glycerol in 1 × PBS buffer]. The purified DtenPHY1 protein was concentrated with Amicon Ultra 15 mL Centrifugal Filters (30,000 MWCO; Millipore). DtenPHY1 protein expressed in *Escherichia coli* LMG194/pPL-PCB was purified as described previously (Duanmu et al., 2014).

### Zinc-Dependent Fluorescence Assay

Purified DtenPHY1 protein from Chlamydomonas CC400 cells was incubated with assembly reaction buffer [20 mM TES, pH 8.0; 0.5 mM EDTA, 1 mM TCEP, 20 mM PCB, 20 mM KCl, 8% (v/v) Glycerol] at room temperature for ∼2 h in the darkness. The mixture was added with SDS loading buffer (no boiling), separated by SDS–PAGE and proteins were transferred to PVDF membrane. PVDF membrane was incubated with 1.3 M zinc acetate for at least 1 h at room temperature. Membrane was washed with ddH_2_O repeatedly and fluorescence signal was visualized by an Odyssey CLx Infrared Imaging System with 700 nm fluorescence channel (LI-COR). Two-fold serial dilutions of DtenPHY1 protein purified from *E. coli* LMG194/pPL-PCB cells were used as positive controls.

### Artificial microRNA-Mediated Gene Silencing

We exploited artificial microRNAs (amiRNAs) to knock-down *PCYA1* gene expression based on previous publications (Molnar et al., 2009). One amiRNA targeting the first exon of *PCYA1* gene was designed by WMD3^4^, with the following sequences: 5′-TCAATTGATTTGGGGATGCTA-3′. Primers for this amiRNA (5′-ctagtTAGCATCCCCAAATCGGTTGAtctcgctgatcggcaccatgggggtggtggtgatcagcgctaTCAATTGATTTGGGGATGCTAg-3′ and 5′-ctagcTAGCATCCCCAAATCAATTGAtagcgctgatcaccaccacccccatggtgccgatcagcgagaTCAACCGATTTGGGGATGCTAa-3′) were annealed by boiling at 100°C and cooled gradually overnight, and then cloned into pChlamiRNA3int vector predigested by SpeI to generate the final construct pChlamiRNA3int-CrPCYA1. The linearized plasmid by KpnI and NotI double digestion was transformed into wild-type strain 4A+ via electroporation (BTX Gemini X2 System, 800 V voltage, 1575 Ω resistance and 50 μF capacitance, 10.0 s pulses interval) and transgenic colonies were selected on TAP agar plates supplemented with 20 μg/mL paromomycin under constant low light (∼30 μmol photons m^-2^s^-1^).

### Protein Extraction and Immunoblot Analysis

Total protein extraction and immunoblot analyses were performed as described previously (Duanmu et al., 2013). Antibodies against GST-tag (66001-I-1g, anti-mouse, 1:2000 dilution) and MBP-tag (66003-I-1g anti-mouse, 1:2000 dilution) were purchased from Proteintech. Antibodies against CrPCYA1 and CrHMOX1 (anti-rabbit, 1:1000 dilution) were generated previously (Duanmu et al., 2013). Antibodies against LHCA1 (AS01 005, anti-rabbit, 1:10000 dilution), PSAD (AS09 461, anti-rabbit, 1:5000 dilution) and PsaA (AS06 172, anti-rabbit, 1:5000 dilution) were purchased from Agrisera. Strep tag antibody (A00626-40, anti-rabbit, 1:4000 dilution) was purchased from GenScript. Antibodies against Actin and Tic40 were from Professor Steven M. Theg (Plant Biology Department, University of California Davis, Davis, CA, United States). The secondary antibody conjugated to horseradish peroxidase (CWBio, CW0102S, goat anti-mouse IgG; or CW0103S, goat anti-rabbit IgG) was used (1:10000 dilution) with the enhanced chemiluminescence detection kit (Bio-Rad, Clarity Western ECL Substrate) for signal acquisition.

### Total RNA Isolation and 3′ RACE

Total RNA from *pcya1-1* was extracted using TransZol plant kit (ET121, Transgen) following the instruction of the kit. RACE (rapid amplification of cDNA ends) was used to amplify the 3′ end cDNA of *pcya1-1* mutant to verify the reading frame of the truncated PCYA1 in *pcya1-1* (Scotto-Lavino et al., 2006). The reverse transcription reaction consisted of 2 μL 5 × reverse transcriptase M-MLV buffer, 0.5 μL reverse transcriptase M-MLV (SD4040, Takara), 0.5 μL Qt primer, 0.5 μL 2.5 mM dNTPs, 0.1 μL RNase inhibitor (SD0316, Takara) and 6.4 μL ddH_2_O (RNase-free) in a total volume of 10 μL. The reaction was incubated at 42°C for 1 h, and the reverse transcriptase M-MLV was inactivated at 75°C for 10 min. The product of reverse transcription reaction was used as template for the first-round amplification using Qo and PCYA1-GSP1 primer pairs. The product of the first-round amplification was subsequently used as template for the second-round amplification using Qi and PCYA1-GSP2 primer pairs. The final product was sequenced using PCYA1-GSP2.

## Results

### Chlamydomonas PCYA1 Contains Unique N-Terminal and C-Terminal Extensions That Exhibit Autoactivation Activity in Yeast Two-Hybrid System

**FIGURE 1:**
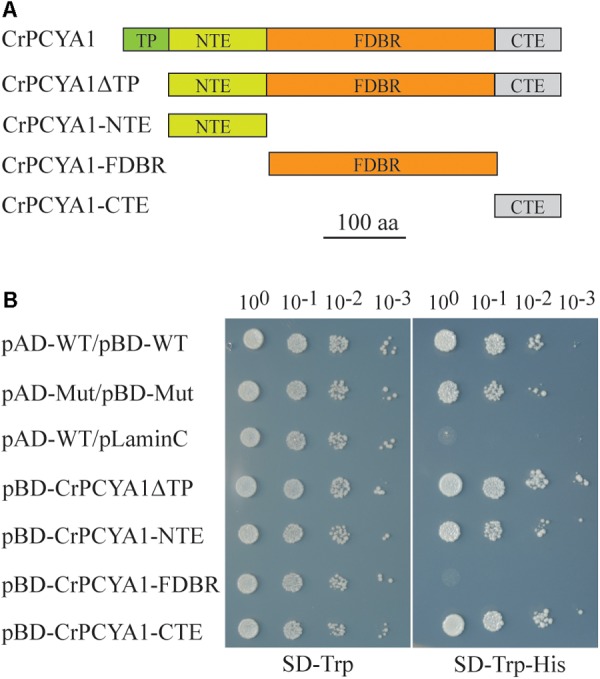
Autoactivation activity test of Chlamydomonas CrPCYA1 protein in yeast. **(A)** Illustration of CrPCYA1 domains and CrPCYA1 truncated proteins used as baits for yeast two hybrid experiment. TP, putative chloroplast transit peptide; NTE, N-terminal extension; FDBR, ferredoxin-dependent biliverdin reductase; CTE, C-terminal extension. **(B)** Growth of yeast cells on synthetic dropout (SD) plates. The coding regions of CrPCYA1 truncated proteins were ligated into pBD-GAL4 vector and transformed into YRG2 yeast strain. Yeast cells were spotted onto SD-Trp and SD-Trp-His agar plates with 10-fold serial dilutions. pAD-WT/pBD-WT and pAD-Mut/pBD-Mut were used as positive controls, and pAD-WT/pLaminC was used as a negative control. WT, Wild-type fragment C of lambda cI repressor (aa 132–236); Mut, E233K mutated fragment of lambda cI repressor (aa 132–236); LaminC, human lamin C (aa 67–230).

CrPCYA1 is a key enzyme involved in bilin biosynthesis in Chlamydomonas (Duanmu et al., 2013). Besides the putative chloroplast transit peptide (TP) and the conserved ferredoxin-dependent biliverdin reductase (FDBR) domain, Chlamydomonas CrPCYA1 contains additional N-terminal extension (NTE) and C-terminal extension (CTE), approximately 120 and 108 amino acids, respectively (**Figure 1A**). It has been well established that FDBR is essential for bilin production in oxygenic photosynthetic organisms (Dammeyer and Frankenberg-Dinkel, 2008). However, there are no reports about the function of additional domains besides FDBR. Compared to homologous sequences of other species including plants, prasinophyte, rhodophyte, glaucophyte and cyanobacteria, the NTE and CTE domains are only found in another chlorophyte alga *V. carteri*, the closest relative of Chlamydomonas. Interestingly, another prasinophyte alga *Micromonas pusilla* CCMP1545 only possesses the CTE domain (**Supplementary Figure S1**). Notably, both NTE and CTE domains of CrPCYA1 exhibited autoactivation activity in yeast, whereas the FDBR domain cannot activate His gene expression (**Figure 1B**).

### Chlamydomonas PCYA1 FDBR Interacts With LPOR but Not DPOR

**FIGURE 2:**
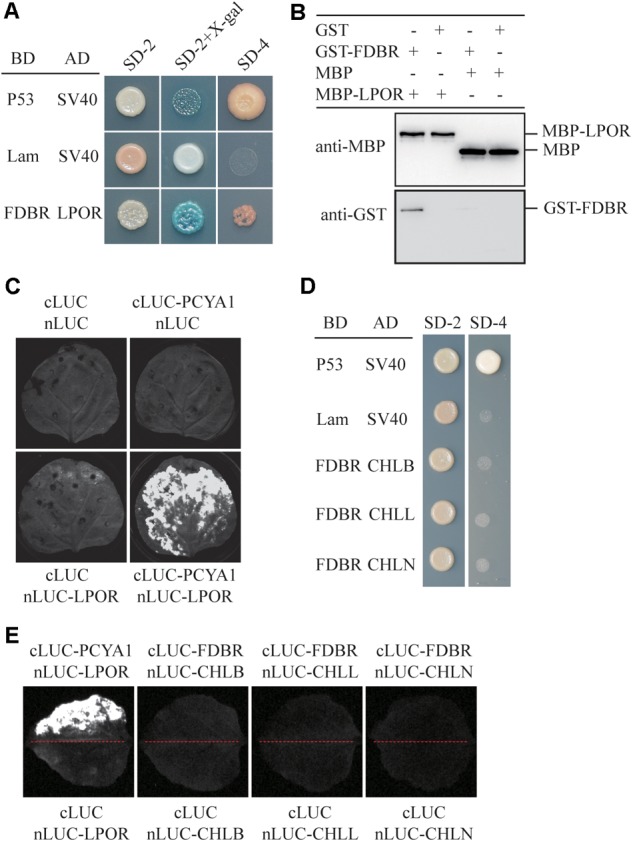
CrPCYA1 FDBR domain interacts with LPOR, but not with DPOR *in vitro* and *in planta*. **(A)** Yeast two-hybrid assay of the interaction between FDBR and LPOR. P53 and SV40 were used as a pair of positive control. Lam and SV40 were used as a negative control. SD-2, SD-Trp-Leu; SD-2+X-gal, SD-Trp-Leu medium supplemented with X-β-gal (80 μg/ml); SD-4, SD-Trp-Leu-His-Ade. **(B)** Pull-down assay for the interaction of FDBR with LPOR *in vitro*. MBP-fusion protein and GST-fusion protein were co-incubated with MBP resin. After washing, proteins captured by MBP resin were analyzed. Antibodies against GST-tag and MBP-tag were used for immunoblotting analysis. **(C)** Split-luciferase assay by co-expression of cLUC-PCYA1 and nLUC-LPOR in tobacco leaves. cLUC and nLUC were used as a negative control. Images were taken at 48 h after infiltration. The strong luminescence signal indicates interaction between PCYA1 and LPOR *in planta*. **(D)** Yeast two-hybrid assay of the interaction between FDBR and three subunits of DPOR: CHLB, CHLL and CHLN. **(E)** Split-luciferase assay for the interaction of FDBR with CHLB, CHLL and CHLN. Co-expression of cLUC and nLUC, cLUC-FDBR and nLUC, cLUC and nLUC-CHLB/L/N in tobacco leaves were used as negative controls. Co-expression of cLUC-PCYA1 and nLUC-LPOR was used as a positive control.

To identify putative PCYA1-interacting proteins, FDBR domain was used as the bait to screen a Chlamydomonas cDNA library and a potential FDBR-interacting protein. Light-dependent NADPH:protochlorophyllide oxidoreductase (LPOR, Cre01.g015350) was thus identified (**Figure 2A**). LPOR is responsible for catalyzing the reaction of protochlorophyllide (PChlide) to chlorophyllide (Chlide), an essential step of chlorophyll biosynthesis (Gabruk and Mysliwa-Kurdziel, 2015). To further verify the interaction between FDBR and LPOR, pull down assay was performed by respective expression of the two proteins as GST-FDBR and MBP-LPOR fusion proteins in *E. coli*. MBP resin was used to capture the MBP tag and associated proteins. We observed that GST-FDBR, but not GST tag, was captured by MBP-LPOR, suggesting the interaction between FDBR and LPOR *in vitro* (**Figure 2B**). Moreover, split luciferase complementation assay identified strong luminescence from *Nicotiana benthamiana* leaves inoculated simultaneously with cLUC-PCYA1 and nLUC-LPOR (**Figure 2C**).

Distinct from higher plants, Chlamydomonas also contains a dark-operative protochlorophyllide oxidoreductase (DPOR) that consists of three chloroplast genes: *ChlB, ChlL* and *ChlN* (Gabruk and Mysliwa-Kurdziel, 2015). To investigate whether FDBR interacts with DPOR, we cloned the three subunit genes into pGADT7- or nLuc- vectors. Both yeast two-hybrid and split luciferase complementation assay were unable to detect interaction between FDBR and CHLB, CHLL or CHLN (**Figures 2D,E**). These data suggest that PCYA1 may specifically regulate the biosynthesis of Chl by interacting with LPOR, but not DPOR in Chlamydomonas.

### Arabidopsis FDBR Homologous Protein HY2 Does Not Interact With PORs

**FIGURE 3:**
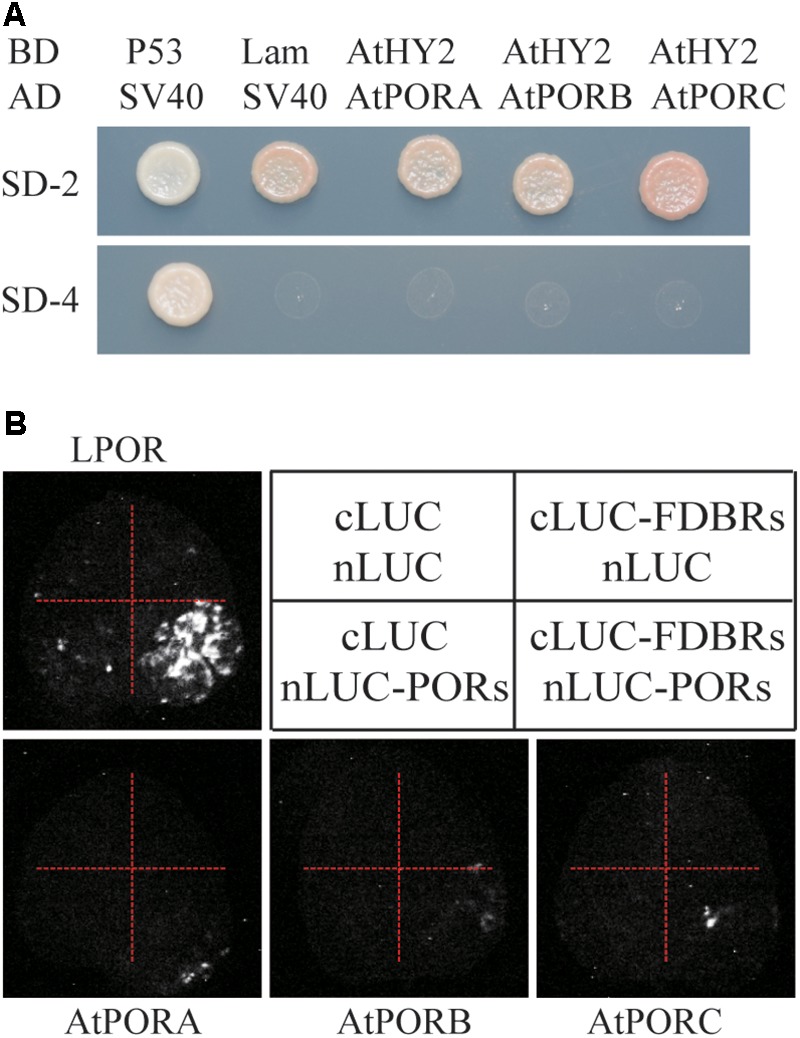
Arabidopsis FDBR protein AtHY2 does not interact with Arabidopsis PORs. **(A)** Yeast two-hybrid assay for the interaction of AtHY2 with AtPORA, AtPORB and AtPORC. Putative chloroplast transit peptides of these chloroplast-localized proteins were removed. **(B)** Split-luciferase assay for the interaction of AtHY2 with AtPORA, AtPORB, and AtPORC (lower three images). Co-expression of cLUC and nLUC, cLUC-AtHY2 and nLUC, cLUC and nLUC-AtPORA/AtPORB/AtPORC in tobacco leaves were used as negative controls. Co-expression of cLUC-PCYA1 and nLUC-LPOR was used as a positive control (upper image).

To evaluate whether the interaction between FDBR and LPOR is unique in Chlamydomonas or conserved also in higher plants, we analyzed their homologous proteins in Arabidopsis. As reported previously, the FDBR in Arabidopsis is encoded by the *HY2* locus and responsible for phytochromobilin biosynthesis from biliverdin IXα (Kohchi et al., 2001). Unlike Chlamydomonas, Arabidopsis lacks DPOR but contains three POR isoenzymes encoded by nuclear genes *PORA, PORB*, and *PORC*, respectively (Gabruk and Mysliwa-Kurdziel, 2015). Chloroplast transit peptides of these four proteins were removed and AtHY2 with AtPORA, AtPORB or AtPORC were co-expressed in yeast cells or tobacco leaves. Our data from Y2H and split luciferase assay indicate no interactions between HY2 and PORs in Arabidopsis (**Figures 3A,B**), further underscoring the specific interaction between FDBR and LPOR in Chlamydomonas.

### PCYA1 Is Partially Associated With Chloroplast Envelope Membrane

**FIGURE 4:**
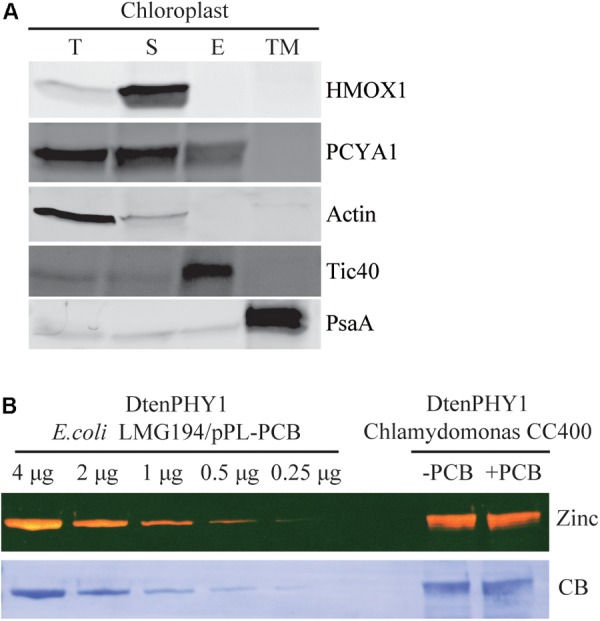
Subcellular distribution of CrPCYA1 in chloroplast and the export of phycocyanobilin to cytosol. **(A)** Biochemical fractionation of chloroplast components. Antibodies against compartmental marker proteins distributed in different chloroplast fractions were employed to distinguish the presence of CrPCYA1 via immunoblot analysis of total proteins (T), soluble fraction (S), envelope membrane (E) and thylakoid membrane (TM) from isolated chloroplast. Tic40, chloroplast envelope marker; PsaA, thylakoid membrane marker. **(B)** Zinc-dependent fluorescence assay. Photosensory core module (PCM) region of the prasinophyte algal DtenPHY1 purified from Chlamydomonas CC400 covalently binds phycocyanobilin (PCB). Upper panel, zinc blot; lower panel, Coomassie blue stain (CB). The two-fold serial dilutions of *E. coli* LMG194/pPL-PCB expressed and purified DtenPHY1 were used as positive controls. –PCB, without addition of PCB; +PCB, assembly with PCB in *vitro*.

Previous research has shown that both HMOX1 and PCYA1 are localized to chloroplast. HMOX1 is a soluble protein, whereas PCYA1 is partially associated with membrane fraction (Duanmu et al., 2013). To determine with which specific membrane fraction PCYA1 is associated, intact chloroplasts of Chlamydomonas cell wall deficient strain CC400 were isolated and further separated into soluble fraction, chloroplast envelope and thylakoid membrane. Immunoblot analyses using envelope membrane marker protein Tic40 and thylakoid membrane marker PsaA indicate the high quality and free of cross-contamination of these two membrane fractions (**Figure 4A**). Consistent with previous observations, HMOX1 was totally soluble and absent from the membrane fractions and majority of PCYA1 was enriched in stromal fraction (Duanmu et al., 2013). However, a small fraction of PCYA1 was also found in chloroplast envelope but none was associated with thylakoid membrane. These data suggest that PCYA1 is dually localized in chloroplast stroma as well as the envelope membrane (**Figure 4A**).

### Phycocyanobilin Is Exported to the Cytosol in Chlamydomonas

Biosynthesis of phycocyanobilin (PCB) in Chlamydomonas has been well established by expression and purification of a cyanobacterial GAF (cGMP-specific phosphodiesterases, adenylyl cyclases and formate hydrogen lyase) domain as the bilin-binding reporter (Duanmu et al., 2013). Since translocation out of the chloroplast is an essential character of *bona fide* chloroplast retrograde signaling molecules, we expressed another bilin-binding protein, the photosensory core module (PCM) of a phytochrome from the marine alga *Dolichomastix tenuilepis* (DtenPHY1) as described previously (Duanmu et al., 2014), in the cytosol of Chlamydomonas wild-type strain CC400. The purified protein exhibits strong fluorescence signal by zinc-dependent assay, comparable to the DtenPHY1 purified from *E. coli* LMG194 engineered to produce PCB cofactor, indicating presence of the covalently bound bilin chromophore of the heterologously expressed protein (**Figure 4B**). Thus, PCB is able to be exported to the cytosol in Chlamydomonas.

### CTE Domain of PCYA1 Is Dispensable for Phototrophic Growth and Photosynthetic Proteins Accumulation

**FIGURE 5:**
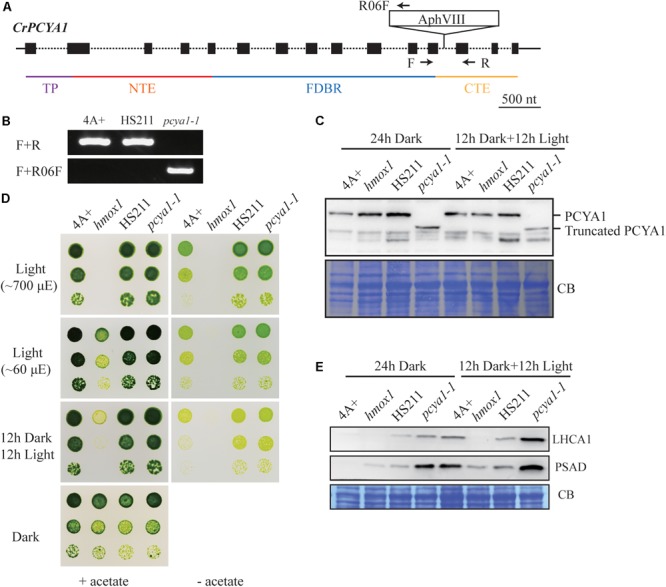
Characterization of the CTE-disrupted *pcya1-1* mutant. **(A)** Schematic diagram of an AphVIII insertion cassette into the *PCYA1* locus in the *pcya1-1* mutant. Black solid line represents 5′UTR and 3′UTR; black dotted line symbolizes intron; black box stands for exon and black arrows indicate the targeting sites of PCR primers. Purple, red, blue, and orange solid lines represent TP, NTE, FDBR and CTE regions of PCYA1, respectively. **(B)** PCR confirmation of the AphVIII insertion in *pcya1-1*. Genomic DNA was extracted from 4A+, HS211 and *pcya1-1*, respectively. PCR were performed using primers depicted in **(A)**. HS211, the parental strain of *pcya1-1*. **(C)** Detection of CrPCYA1 expression in the dark or under light (∼160 μmol photons m^-2^s^-1^). Upper, immunoblot analysis of CrPCYA1 protein accumulation using antibody against CrPCYA1 mature protein; lower, Coomassie blue stain as an equal loading control. **(D)** Comparison of phototrophic growth phenotypes of *pcya1-1* with 4A+, *hmox1* and HS211 under constant low light (∼60 μmol photons m^-2^s^-1^), high light (∼700 μmol photons m^-2^s^-1^) and 12 h dark/12 h light cycle. **(E)** Accumulation of the photosystem I related proteins in *pcya1-1*, 4A+, HS211 and *hmox1* in the dark or under light (∼160 μmol photons m^-2^s^-1^). Immunoblot analyses were performed with LHCA1 and PSAD antibodies. Coomassie blue stain (CB) as an equal loading control. Approximately 30 μg total proteins were loaded in each lane.

Previous results have shown that blocking of bilin biosynthesis in the *hmox1* mutant, or diverging of bilin biosynthesis by chloroplast expression of a mammalian biliverdin reductase both resulted in phototrophic growth deficiency under light (Duanmu et al., 2013). One recently published paper further suggests that accumulation of photosynthetic proteins, especially PSI and LHCI, are drastically affected in the *hmox1* mutant (Wittkopp et al., 2017). Since HMOX1 and PCYA1 are involved in sequential conversion of heme to BV, and then to bilin, we attempted to isolate *PCYA1* mutants and characterize their phenotype. Indeed, we found 2 putative *pcya1* insertional mutants in the Chlamydomonas Stock Center (LMJ.SG0182.002607 and LMJ.RY0402.076336). However, immunoblot analyses indicate none of them have the *PCYA1* disrupted (data not shown). Instead, we obtained an insertional mutant of *PCYA1*, marked as *pcya1-1* from a recently released mutant library containing ∼150,000 insertional mutants (Cheng et al., 2017). The AphVIII insertion cassette in *pcya1-1* mutant is in the 12th intron of *PCYA1* locus (**Figure 5A**) and this insertion was confirmed by genomic DNA extraction and PCR using gene and plasmid insert specific primers (**Figure 5B**). Immunoblotting analysis demonstrates that *pcya1-1* harbors a truncated PCYA1 protein as a result of the eliminated CTE domain (**Figure 5C**). 3′RACE and sequencing results further confirmed the modified reading frame of the truncated PCYA1 protein with CTE disrupted in *pcya1-1* mutant (**Supplementary Table S2**).

To assess the photosynthetic phenotype of *pcya1-1*, we examined the photoautotrophic growth of this mutant under constant low light (∼60 μmol photons m^-2^s^-1^), constant high light (∼700 μmol photons m^-2^s^-1^) and light/dark diurnal cycle conditions. In all experimental settings, phototrophic growth of *pcya1-1* is similar to its parental strain HS211 and the wild-type 4A+, whereas *hmox1* mutant exhibits a photosynthetic deficiency phenotype as reported before (**Figure 5D**) (Duanmu et al., 2013; Wittkopp et al., 2017). Consistently, the Chl *a/b* ratios of *pcya1-1* mutant were similar as its parental strain HS211, whereas the Chl *a/b* ratio of *hmox1* was dramatically decreased under photoautotrophic growth conditions (**Supplementary Figure S2**).

As recently reported, the *hmox1* mutant displays a reduced PSI activity, due to the dramatically reduced or no accumulation of PSI related proteins such as PSAD and LHCA1 during dark to light transitions (Wittkopp et al., 2017). To evaluate the accumulation of these marker proteins, 4A+, HS211, *hmox1* and *pcya1-1* cells were cultured under similar conditions (24 h Dark or 12 h dark followed by 12 h illumination under ∼160 μmol photons m^-2^s^-1^) as described previously (Wittkopp et al., 2017). Total proteins of all strains from above cultures were extracted and subjected to immunoblot analyses. Consistent with previous observations, the LHCA1 protein accumulation in *hmox1* is undetectable and PSAD is drastically reduced relative to 4A+. Compared to the lower-level accumulation of LHCA1 and PSAD in 4A+, HS211 and *pcya1* after 24 h dark acclimation, the illuminated cells demonstrate an obviously increased abundance of these two proteins (**Figure 5E**). Moreover, *pcya1-1* exhibits a higher-level of these PSI marker proteins than HS211 or 4A+ under those two conditions (**Figure 5E**). These results indicate that the CTE domain of PCYA1 is not required for accumulation of representative photosynthetic proteins and phototrophic growth of Chlamydomonas.

### Efficient *PCYA1* Knockdown by Artificial microRNA Does Not Impact Algal Phototrophic Growth

Artificial microRNA-mediated gene silencing has been successfully used to inhibit gene expression and for functional analysis in Chlamydomonas (Vidal-Meireles et al., 2017). Since we have not been able to identify loss-of-function mutants of *PCYA1* based on several available mutant libraries, we attempted to generate *PCYA1* knockdown mutants by the artificial microRNA methodology (Molnar et al., 2009). AmiRNAs for *PCYA1* were designed based on WMD3 tool^5^. One appropriate amiRNA targeting first exon of *PCYA1* gene was selected, constructed into the amiRNA vector and used to transform the wild-type strain 4A+.

**FIGURE 6:**
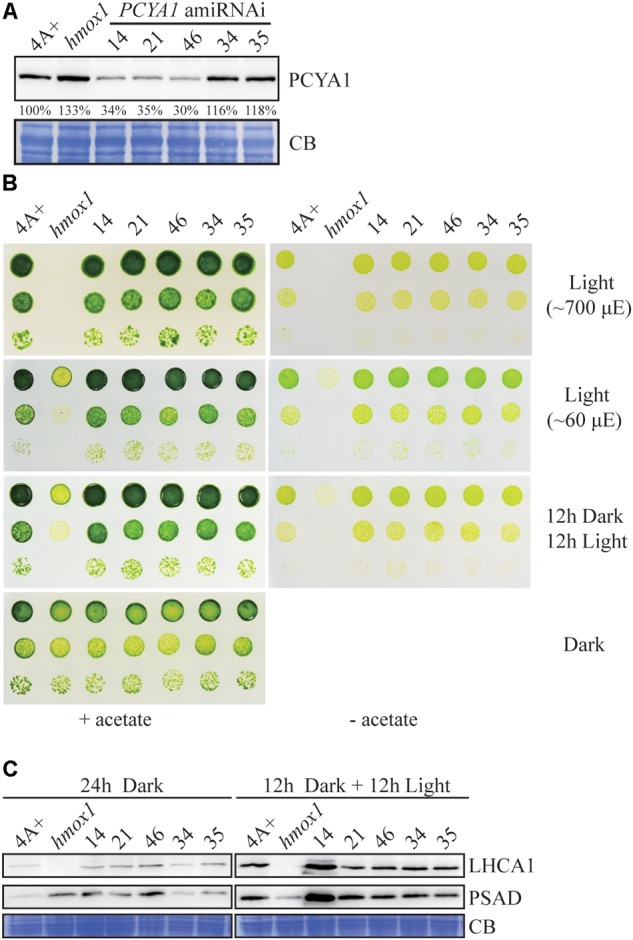
*PCYA1* knockdown by artificial microRNA. **(A)** Analysis of CrPCYA1 protein accumulation in artificial microRNA-mediated *PCYA1* knockdown lines. **(B)** Phototrophic growth of *PCYA1* knockdown mutants were compared with 4A+ and *hmox1* under constant low light, high light and dark/light cycle. Low light, ∼60 μmol photons m^-2^s^-1^; High light, ∼700 μmol photons m^-2^s^-1^. **(C)** Accumulation of representative photosystem I related proteins in *PCYA1* knockdown mutants, 4A+ and *hmox1* in the dark or under light (∼160 μmol photons m^-2^s^-1^). LHCA1 and PSAD antibodies were used for immunoblotting analyses. Around 30 μg total proteins were loaded in each lane.

Several transgenic lines with significantly reduced PCYA1 protein accumulation were isolated, with the protein abundance ranging ∼30–40% of WT level in lines 14, 21, and 46, compared to the near WT level of lines 34 and 35 that were included as controls (**Figure 6A**). Photoautotrophic growth of lines 14, 21, and 46 under different light conditions is indistinguishable from 4A+ WT cells (**Figure 6B**). Examination of LHCA1 and PSAD accumulation also indicates no decrease of these proteins in the amiRNAi lines (**Figure 6C**). The observed higher abundance of LHCA1 and PSAD proteins in line 14 may result from unknown mutations due to the random insertion of the construct. Nonetheless, these results reveal that residual amount of PCYA1 protein is still sufficient to support the photosynthetic proteins accumulation and phototrophic growth of Chlamydomonas.

## Discussion

Chlorophyll biosynthesis is under elaborate control to minimize accumulation of highly phototoxic porphyrin intermediates (Bröcker et al., 2012; Wang and Grimm, 2015). One rate-limiting step of Chl branch is the conversion from PChlide to Chlide that is catalyzed by PORs (Reinbothe et al., 2010). Two types of PORs, namely LPOR and DPOR with distinct evolutionary origin, are found in photosynthetic organisms (Hunsperger et al., 2015). LPOR is light-inducible and present in oxygenic phototrophs, whereas the dark-operative and light-inhibitory DPOR is oxygen-sensitive and encoded by chloroplast genome, and arises from anoxygenic phototrophs (Gabruk and Mysliwa-Kurdziel, 2015; Hunsperger et al., 2015). Apparently DPOR is gradually lost during evolution, since all flowering angiosperm plants do not contain the homologs and thus cannot synthesize Chl in the dark (Hunsperger et al., 2015). In this study, the observed interaction of Chlamydomonas PCYA1 with LPOR, but not with DPOR, is consistent with the hypothesized function of bilin biosynthesis in the regulation of Chl biosynthesis under light, but not in the dark, since the *hmox1* mutant or BVR overexpression lines do not exhibit Chl deficiency phenotype in the dark (Duanmu et al., 2013; Wittkopp et al., 2017).

**FIGURE 7:**
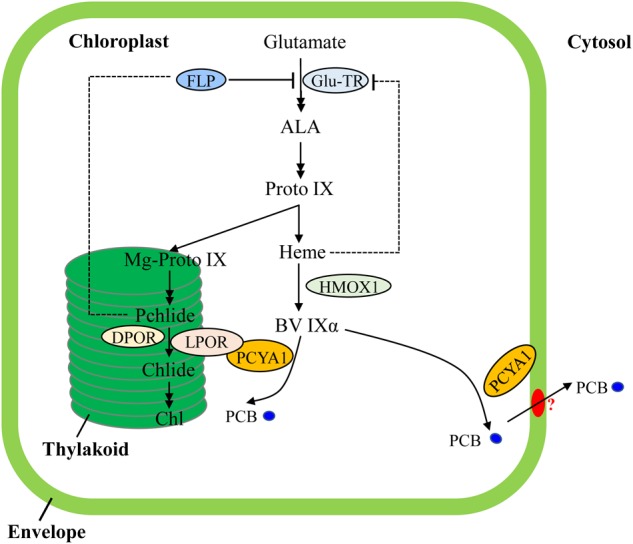
Schematic illustration of tetrapyrrole pathway and regulatory mechanisms of tetrapyrrole biosynthesis in *C. reinhardtii*. FLP is a negative regulator of Glu-TR activity. Heme may also feedback inhibit Glu-TR activity, but to a lesser extent. PCYA1 is likely involved in regulation of Chl biosynthesis by direct interaction with LPOR. Additionally, the PCB molecule is exported from chloroplast to cytosol, via yet-to-be identified bilin transporter (red oval). ALA, 5-Aminolevulinic acid; Proto IX, protoporphyrin IX; Mg-Proto IX, magnesium-protoporphyrin IX; Pchlide, protochlorophyllide; Chlide, chlorophyllide; Chl, chlorophyll; BV IXα, biliverdin IXα; PCB, phycocyanobilin; Glu-TR, Glutamyl-tRNA reductase; HMOX1, heme oxygenase 1; PCYA1, phycocyanobilin ferredoxin-dependent biliverdin oxidoreductase; LPOR, light-dependent protochlorophyllide oxidoreductase; DPOR, light-independent protochlorophyllide oxidoreductase.

Transcriptional regulation of PORs is mainly mediated by phytohormones (ethylene, gibberellin) and light in plants (Masuda and Takamiya, 2004; Gabruk and Mysliwa-Kurdziel, 2015). Chl biosynthesis could be also feedback regulated by heme in Arabidopsis (Terry and Smith, 2013). In contrast, transcriptomic analyses of Chlamydomonas bilin biosynthesis deficient *hmox1* mutant and *in vitro* BV feeding have shown that LPOR transcript abundance remains unchanged and heme inhibition is not the main cause for Chl deficiency (**Figure 7**), indicating distinct types of tetrapyrrole/chlorophyll biosynthesis regulation in chlorophyte algae compared with land plants (Duanmu et al., 2013; Rochaix, 2013; Wittkopp et al., 2017).

Notably, Arabidopsis employs FLU and Chlamydomonas employs FLP (FLU-like protein) as negative feedback regulators to inhibit enzymatic activity of glutamyl-tRNA reductase (GluTR), which is essential for the synthesis of the committed precursor ALA (Meskauskiene et al., 2001; Falciatore et al., 2005). In Arabidopsis, the FLU protein complex containing CHL27 and two PORs isoenzymes (PORB and PORC) can interact with GluTR after PChlide binding to PORs in the dark, resulting in inhibition of Chl biosynthesis (Kauss et al., 2012). Similarly, Chlamydomonas FLP protein also interacts with GluTR and represses Chl biosynthesis (Falciatore et al., 2005). It has not been investigated whether or not Chlamydomonas FLP interacts with LPOR. Since the dark-adapted Chlamydomonas cells should theoretically contain much lower level of PChlide due to a functional DPOR, it is reasonable to hypothesize that the observed PCYA1-LPOR interaction may contribute significantly to the regulation of Chl biosynthesis in the light, or during diurnal transition from dark to light. However, further research should be conducted to investigate whether the PCYA1-LPOR interaction and the bilin or PChlide molecules are also involved in FLP-dependent regulation of GluTR activity in Chlamydomonas. Additionally, it was proposed that LPOR adopts a structure to quench singlet oxygen and the triplet states of PChlide (Reinbothe et al., 2010). It would thus be interesting to test if PCYA1 and bilins are involved in this reaction.

As one of the few enzymes that require light for activation, largely due to the need for its substrate PChlide to absorb light and induce catalytic activity of the POR enzyme, the quantum yields of red light has been shown to be 3∼7 times more effective than blue light in the photoconversion of Pchlide to Chlide for plant POR enzymes (Hanf et al., 2012; Gabruk and Mysliwa-Kurdziel, 2015). Since the longer wavelength light, i.e., red light, is significantly attenuated in water column, more abundant blue or even shorter wavelength light could be more efficiently perceived by aquatic algae for light sensing and photoacclimation (Duanmu et al., 2014; Rockwell et al., 2014a; Wittkopp et al., 2017). In this regard, bilin-dependent blue light-regulated photosensory systems were proposed to regulate photosystem cofactors biosynthesis (i.e., Chl and naphthoquinone) and PSI/LHCI protein accumulation in Chlamydomonas (Wittkopp et al., 2017). PCYA1 with the associated enzymatic product, PCB, may also constitute an essential part of these systems and can significantly enhance LPOR activity under blue light (**Figure 7**). The transcriptional control is essential for long-term regulation of tetrapyrrole/Chl biosynthetic genes. In contrast, this type of post-translational regulatory mechanisms could enable a rapid response during dark/light transitions that accurately balance the enzymatic activities and orchestrate the flow of metabolites for photoacclimation (Czarnecki and Grimm, 2012; Duanmu et al., 2017).

Bilins have been hypothesized to play at least two types of complementary functions in Chlamydomonas, i.e., as chromophore cofactor of putative chlorochrome in the chloroplast to sustain a robust photosynthesis and as retrograde signals to detoxify ROS during dark-to-light transition (Duanmu et al., 2017). The observed subcellular distribution pattern of bilin biosynthetic enzyme PCYA1 is consistent with the dual functions of bilins in Chlamydomonas. Chloroplast envelope associated PCYA1 may support efficient translocation of PCB to the cytosol by interacting with unknown bilin-transporters (Duanmu et al., 2017). Previous studies of oat seedlings also observed the association of FDBR with membrane fraction, congruent with the necessity of bilin export to the cytosol and assembly with apo-phytochromes in plants (McDowell and Lagarias, 2001). Since Chlamydomonas PCYA1 contains unique NTE and CTE that are absent from plant homologs, the NTE and CTE domains may thus not be essential for membrane association. Chl accumulation and phototrophic growth of the *pcya1-1* mutant with CTE disrupted is indistinguishable from wild-type strain, further calling into question the biological functions of this extra domain in Chlamydomonas.

Both the CTE-disrupted *pcya1-1* mutant and knock-down RNAi lines exhibit no obvious defects on phototrophic growth and photosynthetic proteins accumulation. An explanation for the indistinguishable phenotypes of these mutants is that the core FDBR domain in *pcya1-1* mutant and the residual amount of PCYA1 protein in RNAi lines are still sufficient to sustain bilin biosynthesis and maintain the biological functions. The inability to obtain a *pcya1* null mutant may underscore the indispensable roles of this enzyme in Chlamydomonas. Indeed, investigations of *PCYA* in cyanobacterium *Synechococcus sp*. strain PCC 7002 demonstrated that this gene was unable to be inactivated in wild-type cells, but could be easily disrupted after introducing the Arabidopsis phytochromobilin synthase HY2 into the cell, indicating the requirements of PCYA and/or the bilins for cell survival (Alvey et al., 2011).

## Conclusion

In conclusion, the discovery reported in this study has provided novel insights into the multifaceted biological functions of bilins and bilin biosynthetic enzyme in Chlamydomonas. However, many questions still remain to be answered. Are the interactions between PCYA1 and LPOR dynamically regulated in the dark versus under light? What are the biological roles of bilins and PChlide in fine-tuning this interaction and regulating LPOR activity, and whether or not this type of post-translational regulation of Chl biosynthesis is conserved in all DPOR-containing or phytochrome-less phototrophs, including eukaryotic green/red algae and gymnosperms? Construction and characterization of *pcya1* mutants with NTE deletion or the whole gene disrupted by either random mutagenesis or by recently established CRISPR technology in Chlamydomonas (Ferenczi et al., 2017; Greiner et al., 2017) should be able to greatly deepen our understanding of the bilin-mediated tetrapyrrole/Chl biosynthetic regulation and photoacclimation in chlorophyte algae and other photosynthetic organisms.

## Author Contributions

WZ and DD designed the research and analyzed the results. WZ, HZ, and HL performed the experiments. XD and KH constructed and analyzed the mutant library. WZ, HZ, and DD wrote the paper. All the authors read and approved the manuscript.

## Conflict of Interest Statement

The authors declare that the research was conducted in the absence of any commercial or financial relationships that could be construed as a potential conflict of interest.

## Footnotes

1 https://phytozome.jgi.doe.gov/pz/portal.html

2 http://www.clustal.org/clustal2/

3 http://www.lynnon.com/dnaman.html

4 http://wmd3.weigelworld.org

5 http://wmd3.weigelworld.org/cgi-bin/webapp.cgi

## Supplementary Material

FIGURE S1 Multiple sequence alignments of FDBRs. CLUSTAL X and DNAMAN tools were used for the alignments. Consensus sequences were marked as lowercase letters. The black, pink and cyan bars indicate 100%, ≥ 75% and ≥ 50% sequence similarity, respectively. The overline regions with purple, red, blue and green solid lines represent TP, NTE, FDBR, and CTE domains of PCYA1 in Chlamydomonas, respectively. Cre, *C. reinhardtii*; Vca, *V. carteri*; Ath, *A. thaliana*; Ppa, *Physcomitrella patens*; Syn7002, *Synechococcus sp.* PCC 7002; Syn6803, *Synechocystis sp.* PCC 6803; Pmi, *Paulinella micropora;* Mpu, *Micromonas pusilla* CCMP1545; Cme, *Cyanidioschyzon merolae* strain 10D; Glo, *Gloeochaete wittrockiana* SAG46.84.

FIGURE S2 Comparisons of Chl *a/b* ratios in 4A+, *hmox1*, HS211 and *pcya1-1*. These strains were grown under constant light (∼160 μE) with (black bar) or without acetate (gray bar). Data show means of three biological replicates ± SD.

TABLE S1 Primers used in this study.

TABLE S2 The amino acid sequences of PCYA1 in wild-type Chlamydomonas and *pcya1-1* mutant. Yellow letters represent the putative chloroplast transit peptide (TP); Red letters, the N-terminal extension (NTE); Cyan letters, the FDBR domain; Green letters, C-terminal extension (CTE). The mutated amino acid sequence of CTE in *pcya1-1* mutant was marked as gray letters.
