# Supplementary material for: Characterization of Ferredoxin-Dependent Biliverdin Reductase PCYA1 Reveals the Dual Function in Retrograde Bilin Biosynthesis and Interaction With Light-Dependent Protochlorophyllide Oxidoreductase LPOR in Chlamydomonas reinhardtii
Source: Front Plant Sci. 2018 May 23;9:676. doi: 10.3389/fpls.2018.00676 (PMC5974162; doi:10.3389/fpls.2018.00676)
Supplement: FIGURE S1 — Multiple sequence alignments of FDBRs. CLUSTAL X and DNAMAN tools were used for the alignments. Consensus sequences were marked as lowercase letters. The black, pink and cyan bars indicate 100%, ≥ 75% and ≥ 50% sequence similarity, respectively. The overline regions with purple, red, blue and green solid lines represent TP, NTE, FDBR, and CTE domains of PCYA1 in Chlamydomonas, respectively. Cre, C. reinhardtii; Vca, V. carteri; Ath, A. thaliana; Ppa, Physcomitrella patens; Syn7002, Synechococcus sp. PCC 7002; Syn6803, Synechocystis sp. PCC 6803; Pmi, Paulinella micropora; Mpu, Micromonas pusilla CCMP1545; Cme, Cyanidioschyzon merolae strain 10D; Glo, Gloeochaete wittrockiana SAG46.84. [file Image_1.PDF]

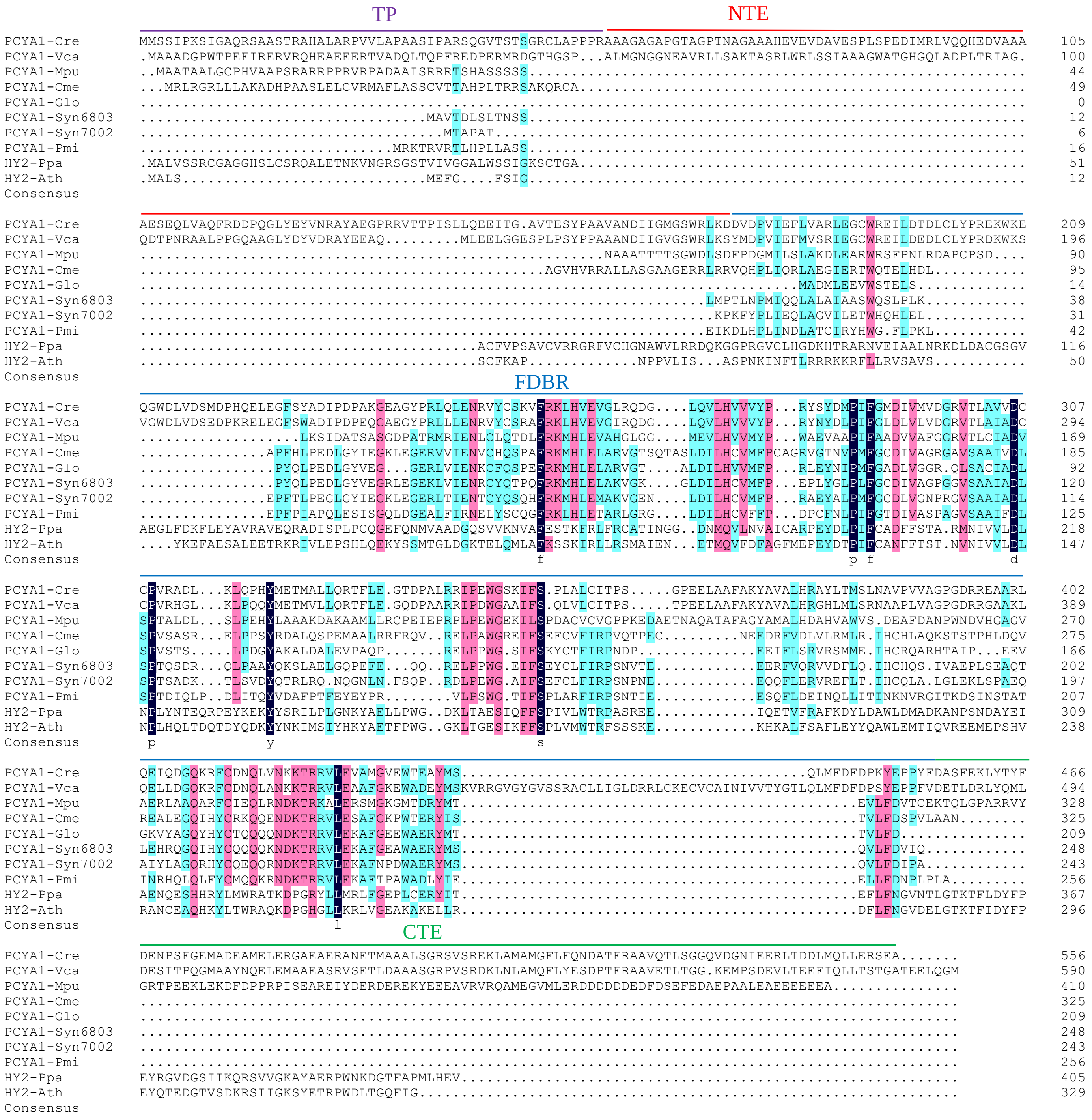
TP
NTE
FDBR
CTE
PCYA1-Cre
PCYA1-Vca
PCYA1-Mpu
PCYA1-Cme
PCYA1-Glo
PCYA1-Syn6803
PCYA1-Syn7002
PCYA1-Pmi
HY2-Ppa
HY2-Ath
Consensus
