# Supplementary material for: Characterization of Ferredoxin-Dependent Biliverdin Reductase PCYA1 Reveals the Dual Function in Retrograde Bilin Biosynthesis and Interaction With Light-Dependent Protochlorophyllide Oxidoreductase LPOR in Chlamydomonas reinhardtii
Source: Front Plant Sci. 2018 May 23;9:676. doi: 10.3389/fpls.2018.00676 (PMC5974162; doi:10.3389/fpls.2018.00676)

Fig S2. Comparisons of Chl *a/b* ratios in 4A+, *hmox1*, HS211 and *pcya1-1*. These strains were grown under constant light (~160 μE) with (black bar) or without acetate (grey bar). Data show means of three biological replicates ± SD.

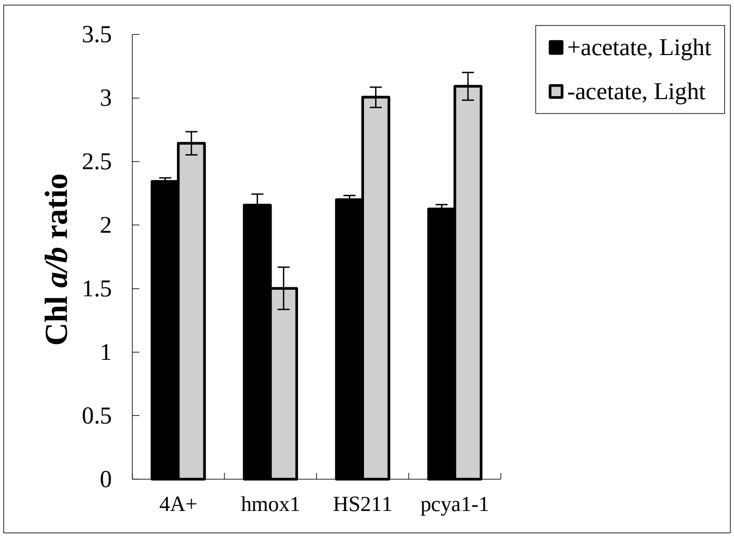

Supplement: FIGURE S2 — Comparisons of Chl a/b ratios in 4A+, hmox1, HS211 and pcya1-1. These strains were grown under constant light (∼160 μE) with (black bar) or without acetate (gray bar). Data show means of three biological replicates ± SD. [file Image_2.PDF]
