# Supplementary material for: Characterization of Ferredoxin-Dependent Biliverdin Reductase PCYA1 Reveals the Dual Function in Retrograde Bilin Biosynthesis and Interaction With Light-Dependent Protochlorophyllide Oxidoreductase LPOR in Chlamydomonas reinhardtii
Source: Front Plant Sci. 2018 May 23;9:676. doi: 10.3389/fpls.2018.00676 (PMC5974162; doi:10.3389/fpls.2018.00676)
Supplement: TABLE S1 — Primers used in this study. [file Table_1.PDF]

Table S1. Primers used in this study

Purpose	Primer name	Primer sequences (5'to 3')
**yeast-two hybrid**	CrLPOR-s-EcoRI	aagaattcCGCGTGATGCCCGCCCCCG
	CrLPOR-as-BamHI	aaggatccTTACGCGCTCAGGCCCACC
	CrFDBR-s-EcoRI	aagaattcGACGTGGACCCGGTCATTGA
	CrFDBR-as-BamHI	aaggatccTTACTCGTACTTGGG GTCAAAG
	CrChlB-s-EcoRI	aggccagtgaattcATGAAATTAGCTTATTGGA
	CrChlB-as-BamHI	gagctcgatggatccTTAAGCCGACAAAGCTTCT
	CrChlL-s-EcoRI	aggccagtgaattcATGAAATTAGCTGTTTACG
	CrChlL-as-BamHI	gagctcgatggatccttaAATTTTAAGATAGAAA
	CrChlN-s-EcoRI	aggccagtgaattcATGTTAGATGGTGCCACA
	CrChlN-as-BamHI	gagctcgatggatccTTAAGAAATAGCATTTACA
	AtHY2-s-EcoRI	ccatggaggccgaattcGTCTCTGCTGTGTCGTAT
	AtHY2-as-BamHI	caggtcgacggatccTTAGCCGATAAATTGTCCTG
	AtPORA-s-EcoRI	ggaggccagtgaattcTGCAAGAGGGAACAGAGCT
	AtPORA-as-BamHI	gagctcgatggatccTTAGGCCAAGCCTACGAG
	AtPORB-s-EcoRI	ggaggccagtgaattcTCCGAACATGGATCTTCCT
	AtPORB-as-BamHI	gagctcgatggatccTTAGGCCAAGCCCACGAG
	AtPORC-s-EcoRI	ggaggccagtgaattcGCACAGACAGTTACAGCCA
	AtPORC-as-BamHI	gagctcgatggatccTCATGCCAAACCAACAAG
**pull down assay**	CrLPOR-s-EcoRI	aagaattcCGCGTGATGCCCGCCCCCG
	CrLPOR-as-BamHI	aaggatccTTACGCGCTCAGGCCCACC
	CrFDBR-s-BamHI	TGTTCCAGGGGCCCCTGggattcGACGTGGACCCGGTCATTG
	CrFDBR-as-NotI	TCAGTCAGTCACGATgcggccgcTTACTCGTACTTGGGGTCA
**split luciferase complementation assay**	CrPCYAΔTP-KpnI-s	aaggtaccATGATGTCGAGCATCCCCAA
	CrPCYAΔTP-BamHI-as	aaggatccACGCCTCACTTCGCTCCAGTA
	CrLPORΔTP-KpnI-s	aaggtaccATGGACTACAAGGACGACGACGACAAAATGGCCCTCACCATGTCCG
	CrLPORΔTP-SalI-as	aagTCGACCGCGCTCAGGCCCACCAGC
	AtHY2-s-KpnI	cgtcccggggcggtaccGTCTCTGCTGTGTCGTAT
	AtHY2-as-BamHI	gtccatttgttggatccAGCCGATAAATTGTCCTGTT
	AtPORA-s-KpnI	aaggtaccATGGACTACAAGGACGACGACGACAAATGCAAGAGGGAACAGAGC
	AtPORA-as-SalI	aagtcgacGGCCAAGCCTACGAGCTT
	AtPORB-s-KpnI	aaggtaccATGGACTACAAGGACGACGACGACAAATCCGAACATGGATCTTCC
	AtPORB-as-SalI	aagtcgacGGCCAAGCCCACGAGCTT
	AtPORC-s-KpnI	aaggtaccATGGACTACAAGGACGACGACGACAAAGCACAGACAGTTACAGCC
	AtPORC-as-SalI	aagtcgacTGCCAAACCAACAAGCTTCTCGCT
	CrFDBR-KpnI-s	aaggtaccGACGTGGACCCGGTCATT
	CrFDBR-BamHI-as	aaggatccACTCGTACTTGGGGTCAAA
	CrChlL-SalI-as	aagtcgacAATTTTAAGATAGAAATCT
	CrChlL-KpnI-s	aaggtaccTGGTCTCACCCACAGTTCGAGAAGggatccATGAAATTAGCTGTTTAC
	CrChlB-SalI-as	aagtcgacAGCCGACAAAGCTTCTTT
	CrChlB-BamHI-s	aaggatccATGAAATTAGCTTATTGG
	CrChlN-SalI-as	aagtcgacAGAAATAGCATTTACAGA
	CrChlN-KpnI-s	aaggtaccATGTGGTCTCACCCACAGTTCGAGAAGggatccATGTTAGATGGTGCCACA
**DtenPHY1 expression in Chlamydomonas**	DtenPHY1-EcoRV-as	aagatatcCGCGGCCAGGCGGGTCAC
	DtenPHY1-NdeI-s	gggaattccatatgAAGATGGCGGCGAAGA
***pcya1* mutant identification**	R06F	GGCGAAGCTTGGTACCGCTA
	F	GCCTCGACAAGGACCCGTGC
	R	CACGGACCGTCCCGACAACG
**artificial microRNA-RNAi**	amiRNA-PcyA1-F3	ctagtTAGCATCCCCAAATCGGTTGATCTCGCTGATCGGCACCATGGGGGTGGTGGTG<br>ATCAGCGCTATCAATTGATTTGGGGATGCTAG
	amiRNA-PcyA1-R3	ctagcTAGCATCCCCAAATCAATTGATAGCGCTGATCACCACCACCCCCATGGTGCCG<br>ATCAGCGAGATCAACCGATTTGGGGATGCTAA
**3'RACE**	Qt	CCAGTGAGCAGAGTGACGAGGACTCGAGCTCAAGCTTTTTTTTTTTTTTTTT
	Qo	CCAGTGAGCAGAGTGACG
	Qi	GAGGACTCGAGCTCAAGC
	PCYA1-GSP1	GAGAGTCACCACGCCGATCAGCT
	PCYA1-GSP2	GTGCCTGTACCCGCGTGAGAAGT
