# Supplementary material for: Characterization of Ferredoxin-Dependent Biliverdin Reductase PCYA1 Reveals the Dual Function in Retrograde Bilin Biosynthesis and Interaction With Light-Dependent Protochlorophyllide Oxidoreductase LPOR in Chlamydomonas reinhardtii
Source: Front Plant Sci. 2018 May 23;9:676. doi: 10.3389/fpls.2018.00676 (PMC5974162; doi:10.3389/fpls.2018.00676)
Supplement: TABLE S2 — The amino acid sequences of PCYA1 in wild-type Chlamydomonas and pcya1-1 mutant. Yellow letters represent the putative chloroplast transit peptide (TP); Red letters, the N-terminal extension (NTE); Cyan letters, the FDBR domain; Green letters, C-terminal extension (CTE). The mutated amino acid sequence of CTE in pcya1-1 mutant was marked as gray letters. [file Table_2.PDF]

Table S2. The amino acid sequences of PCYA1 in wild type Chlamydomonas and *pcya1-1* mutant. Yellow letters represent the putative chloroplast transit peptide (TP); Red letters, the N-terminal extension (NTE); Cyan letters, the FDBR domain; Green letters, C-terminal extension (CTE). The mutated amino acid sequence of CTE in *pcya1-1* mutant was marked as grey letters.

PCYA1 in wild type:

MMSSIPKSIGAQRSAASTRAHALARPVVLAPAASIPARSQGVTSTSGRCLAPPPRAAAGAG
APGTAGPTNAGAAAHEVEVDAVESPLSPEDIMRLVQQHEDVAAAAESEQLVAQFRDDPQ
GLYEYVNRAYAEGPRRVTTPISLLQEEITGAVTESYPAAVANDIIGMGSWRLKDDVDPVIE
FLVARLEGCWREILDTDLCLYPREKWKEQGWDLVDSMDPHQELEGFSYADIPDPAKGEA
GYPRLQLENRVYCSKVFRKLHVEVGLRQDGLQVLHVVVYPRYSYDMPIFGMDIVMVDGR
VTLAVVDCCPVRADLKLQPHYMETMALLQRTFLEGTDPALRRIPEWGSKIFSPLALCITPS
GPEELAAFAKYAVALHRAYLTMSLNAVPVVAGPGDRREAARLQEIQDGQKRFCDNQLVN
KKTRRVLEVAMGVEWTEAYMSQLMFDFDPKYEPPYFDASFEKLYTYFDENPSFGEMADE
AMELERGAEAERANETMAAALSGRSVSREKLAMAMGFLFQNDATFRAAVQTLSGGQVD
GNIEERLTDDLMQLLERSEA*

Truncated PCYA1 in *pcya1-1* mutant:

MMSSIPKSIGAQRSAASTRAHALARPVVLAPAASIPARSQGVTSTSGRCLAPPPRAAAGAG
APGTAGPTNAGAAAHEVEVDAVESPLSPEDIMRLVQQHEDVAAAAESEQLVAQFRDDPQ
GLYEYVNRAYAEGPRRVTTPISLLQEEITGAVTESYPAAVANDIIGMGSWRLKDDVDPVIE
FLVARLEGCWREILDTDLCLYPREKWKEQGWDLVDSMDPHQELEGFSYADIPDPAKGEA
GYPRLQLENRVYCSKVFRKLHVEVGLRQDGLQVLHVVVYPRYSYDMPIFGMDIVMVDGR
VTLAVVDCCPVRADLKLQPHYMETMALLQRTFLEGTDPALRRIPEWGSKIFSPLALCITPS
GPEELAAFAKYAVALHRAYLTMSLNAVPVVAGPGDRREAARLQEIQDGQKRFCDNQLVN
KKTRRVLEVAMGVEWTEAYMSQLMFDFDPKYEPPYFDASFEKLYTYFDENPSFGEMGKS
VCVAGVGGGLLPPQHGGWSAGRVAAAGKSS*
